# The 6th World Symposium on Pulmonary Hypertension: what’s old is new

**DOI:** 10.12688/f1000research.18811.1

**Published:** 2019-06-19

**Authors:** David F Condon, Nils P Nickel, Ryan Anderson, Shireen Mirza, Vinicio A de Jesus Perez

**Affiliations:** 1 Division of Pulmonary and Critical Care Medicine, Stanford University, Stanford, USA; 2 Vera Moulton Wall Center for Pulmonary Vascular Research, Stanford University, Stanford, USA

**Keywords:** pulmonary hypertension, hemodynamics, therapy, genetics

## Abstract

In February 2018, the 6th World Symposium on Pulmonary Hypertension (WSPH) brought together experts from various disciplines to review the most relevant clinical and scientific advances in the field of PH over the last 5 years. Based on careful review and discussions by members of the different task forces, major revisions were made on the hemodynamic definition for various forms of PH and new genes were added to the list of genetic markers associated with pulmonary arterial hypertension (PAH) and pulmonary veno-occlusive disease. In addition, the use of risk stratification tools was encouraged as a strategy to reduce one-year mortality risk in PAH patients through early implementation of PAH therapies. While members of the medical community are still debating some of the proposed changes, the new WSPH guidelines advocate early diagnosis and initiation of combination therapy to reduce mortality and improve quality of life in patients with PH.

## Introduction

Following an epidemic of pulmonary arterial hypertension (PAH) cases in the 1960s in Germany, Austria and Switzerland due to the anorectic stimulant Aminorex, the World Health Organization (WHO) organized the first World Symposium on Pulmonary Hypertension (WSPH) in October 1973 in Geneva, Switzerland ^1, 2^. Forty-five years later, the 6th WSPH took place in Nice and brought together 124 experts in 13 different task forces to review clinical and scientific advances pertinent to improving the diagnosis and management of PH ^3^. Considering the recommendations of the various task forces, the 6th WSPH has revised the hemodynamic definition of PH for the first time since 1973. Newly discovered genes associated with hereditary and idiopathic PAH (HPAH and IPAH, respectively) were discussed and recommendations were made to update the clinical classification and implement risk stratification in the treatment algorithm of PH. These changes are now a topic of active debate within the scientific community. This report will summarize the highlights of the 6th WSPH, and the implications of the proposed changes to future basic and clinical research in PH will also be discussed.

## Advances in genetics and genomics

### Novel genes associated with pulmonary arterial hypertension

Since its discovery in 2000 ^4^, *BMPR2* mutations remain the most common genetic cause of PAH, accounting for about 80% of HPAH and about 20% of IPAH. Besides *BMPR2*, other transforming growth factor beta (TGF-β) superfamily genes, including *ALK1/ACVRL1* (a heterodimeric partner of BMPR2), *BMP9* (a BMPR2 ligand), *ENG* (a co-receptor for BMPR2 signaling), and *SMAD1*, *4*, and *9* (downstream BMP signaling molecules), have been linked to both HPAH and IPAH ^5^. In the last 5 years, next-generation sequencing technologies have been applied to genetic discovery in patient populations with IPAH, HPAH, and drug-induced PAH. For example, whole exome sequencing (WES) in *BMPR2*-negative HPAH patients led to the discovery of two novel genes: *CAV1* (involved in BMPR2 membrane localization and signaling) ^6^ and *KCNK3* (a potassium channel that regulates resting membrane potential) ^7^. More recently, a WES screen of pediatric patients with HPAH revealed that, in addition to *BMPR2*, the most common mutation in this patient population was in *TBX4*, a gene linked to small patella syndrome ^8^. Besides WES, whole genome sequencing (WGS) has now been used to characterize novel high-risk gene variants in large patient populations. A WGS study of 1048 patients with PAH by Gräf and colleagues ^9^ screened and found novel mutations in *GDF2* (which codes for BMP9) and identified *ATP13A3*, *AQP1*, and *SOX17* ^10^ as novel gene candidates, although the specific pathogenic mechanism of these in PAH remains to be determined.

Pulmonary veno-occlusive disease (PVOD) and pulmonary capillary hemangiomatosis (PCH) are severe forms of WHO group 1 PH with a rapidly progressive clinical course and poor response to therapy. In 2014, the first of two scientific groups reported that mutations in *EIF2AK4* (a kinase involved in amino acid metabolism) were associated with both PVOD and PCH ^11, 12^. In contrast to *BMPR2* mutations, *EIF2AK4* mutations are autosomal recessive and completely penetrant. Detection of *EIF2AK4* in a patient with PAH can establish the diagnosis of PVOD/PCH in the appropriate clinical context without necessitating lung biopsy ^12^.

The 6th WSPH Task Force on Genetics and Genomics has updated the list of gene candidates for PAH to include these new genes and strongly recommends research in understanding how they are linked to PAH pathogenesis and how this information could be used in biomarker discovery and new therapeutics ^13^.

### Genetic counseling

As WES and WGS become less expensive and more available, the importance of explaining these results and their significance for patients and potential offspring is critical. Given the complexities of the genetic processes underlying HPAH, this only adds nuance to the difficult task of genetic counseling to patients with a family or personal history of HPAH looking to conceive. The psychosocial concerns of genetic screening for a disease for which there is no prevention and no specific cure may weigh heavily on those affected and may cause guilt to those not phenotypically affected or who could pass disease-causing mutations onto children. After discussing the potential benefits of genetic screening (that is, early detection of family members and earlier initiation of therapy when indicated), the 6th WSPH task force recommended that genetic screening be carried out under the guidance of a genetic counselor or clinical geneticist ^13^. At this point, a pedigree can be generated to identify relatives at risk, although gene testing or screening should be initiated with affected patients. There are different methods of evaluating the genetics of affected patients. In addition to commercially available diagnostic PAH/PVOD gene panels, WES or WGS may be appropriate for a patient with a negative gene panel and also have important relevance for research. However, it must be stressed that these diagnostic tests should be ordered by a specialist who is trained in genetics and who would assume the responsibility of counseling patients and their families regarding the implications of the test results.

## Hemodynamic definition and updated clinical classification of pulmonary hypertension

Since the first WSPH in 1973, PH has been defined as a mean pulmonary artery pressure (mPAP) of at least 25 mm Hg. During the 6th WSPH, the task force members suggested that this definition be changed to mPAP of more than 20 mm Hg ^14^. The authors based this recommendation on the fact that the original definition of mPAP of at least 25 mm Hg was chosen somewhat arbitrarily and does not represent the upper limit of normal mPAP in the general population. Review of all available studies on pulmonary hemodynamics in healthy individuals indicates that a normal mPAP is approximately 14 ± 3.3 mm Hg and that the upper limit (>97.5th percentile) of normal is 20 mm Hg ^15^. Major concerns regarding inclusion of individuals with mPAP between 21 and 24 mm Hg are the risk of PH diagnosis in otherwise healthy individuals and the lack of data that treatment of this population would be safe or beneficial (or both). Despite these concerns, studies published in the past 5 years suggest that individuals with mPAP of 21 to 24 mm Hg are at increased risk of poor outcomes ^16– 18^ and tend to progress to “overt PH (mPAP of at least 25 mm Hg)” more often than patients with mPAP of not more than 20 mm Hg over a 2- to 3-year follow-up ^19, 20^. Additionally, treatment of some individuals with mPAP of 15 to 25 mm Hg in two separate chronic thromboembolic PH cohorts yielded improved clinical results ^21, 22^ and at least one trial is under way in patients with the same mPAP criteria in PAH associated with systemic sclerosis (ClinicalTrials.gov identifier: NCT02290613). Based on these data, the 6th WSPH task force recommended that a new mPAP of more than 20 mm Hg cutoff for diagnosing PH is both clinically warranted and in the best interest of the patient ( Table 1).

**Table 1.** Hemodynamic profiles of pulmonary hypertensio.

Classification	Mean pulmonary artery pressure	Pulmonary capillary wedge pressure	Pulmonary vascular resistance
Isolated pre-capillary PH	>20 mm Hg	<15 mm Hg	>3 WU
Combined pre- and post-capillary PH		>15 mm Hg	>3 WU
Isolated post-capillary PH		>15 mm Hg	<3 WU

The 6th World Symposium on Pulmonary Hypertension defined three hemodynamic profiles of pulmonary hypertension (PH): isolated pre-capillary PH, combined pre- and post-capillary PH, and isolated post-capillary PH. WU, Wood units.

Pulmonary vascular resistance (PVR) has also taken on a new role in PH classification. Established in accordance with the 5th WSPH, PVR was considered important for diagnosing PAH but was not included in the general definition of all forms of PH, and the diagnostic criteria for combined pre- and post-capillary PH remained unclear. Now, in keeping with the 6th WSPH update, all PH (mPAP of more than 20 mm Hg) will be further subclassified as pre-capillary PH (as seen in PAH), isolated post-capillary PH (IpcPH), or combined pre- and post-capillary PH (CpcPH) on the basis of pulmonary arterial wedge pressure (PAWP) and PVR. Although the threshold and application of significant PAWP have not changed (not more than 15 mm Hg in PAH, more than 15 mm Hg in all CpcPH), PVR now defines the presence or absence of pre-capillary PH (PVR of less than 3 Wood units [WU] = IpcPH, PVR of at least 3 WU in PAH and CpcPH) ^14^. The subcategorization and method of detecting CpcPH remain controversial. Evidence is suggestive of CpcPH as a distinct entity from PAH or IpcPH, which carries a different prognosis both before and after heart transplantation (6th WSPH on left heart disease–PH section). While PVR of at least 3 WU has strong evidence to support its diagnostic utility, other hemodynamic markers such as the transpulmonary gradient and pulmonary arterial compliance have demonstrated value in two studies for clarifying the diagnosis, and thus prognosis, in at-risk individuals ^23, 24^. Efforts have been made to standardize methodology to distinguish between CpcPH and IpcPH via exercise testing (CpcPH should have a greater rise in PVR than patients with IpcPH) and fluid challenge (IpcPH should have a disproportionate rise in PAWP), although these are technically difficult in the case of the former and there is a lack of consensus cutoff data for what would characterize an abnormal physiologic response in the latter.

## Risk stratification and medical therapy of pulmonary arterial hypertension

### Risk stratification

The clinical status and trajectory of patients with PAH can be defined through the use of multiple validated risk stratification tools. The many available comprehensive risk stratification tools use a combination of parameters that assess clinical, functional, cardiac, and hemodynamic status to assign patients to low-, intermediate-, or high-risk categories. Without delving into the intricacies of the superiority of one validated assessment tool over the other, what is clear is that the baseline use of these tools can predict survival and event-free survival for up to 5 years ^25, 26^ and that, in certain situations, re-classification into a different category at follow-up on the basis of re-scoring can predict outcomes over the next year. Within the mélange of these risk stratification systems, the following parameters appear to have the greatest predictive capability: functional class, six-minute walk distance (6MWD), N-terminal pro-brain natriuretic peptide/brain natriuretic peptide (NT-proBNP/BNP) levels, cardiac index, right atrial pressure, and mixed venous oxygen saturation (SvO _2_) ^27, 28^. The guidelines acknowledge that the limitations of these tools include the inability to account for non-modifiable/ill-modifiable risk factors such as age, sex, comorbidities, and additional data available through advanced investigative techniques such as cardiac magnetic resonance and cardiopulmonary exercise testing ^29^. In addition, most of these tools are time-consuming to use and require data points that are not routinely obtained. At present, it is also unclear which parameters define the set of patients with prolonged clinical stability. Use of machine learning and artificial intelligence in future work may assist in developing new, low-bias prediction tools that eliminate some of the above pitfalls with current prediction scores and tools ^30, 31^.

Even as these risk calculators continue to evolve, they remain an important tool in assessing patients in the clinical and research setting while helping to guide management. The task force members recommend that future iterations or refinements also take into consideration accuracy and cost-effectiveness of obtaining included parameters ^29^.

### Selected highlights from the updated treatment algorithm

The Dana Point 4th WSPH recommendations from 2008 led to a significant shift in PAH randomized control trial (RCT) design. Multiple recent trials using more clinically meaningful end-points (such as time to clinical worsening, death, or transplant instead of 6MWD) have led to improved confidence in, and adoption of, choices for therapeutic strategy and drug combinations. For all patients with PAH, activity within symptom limits is advised, and recent data affirm the benefits of supervised training across the spectrum of functional impairment ^29^.

**Figure 1.**
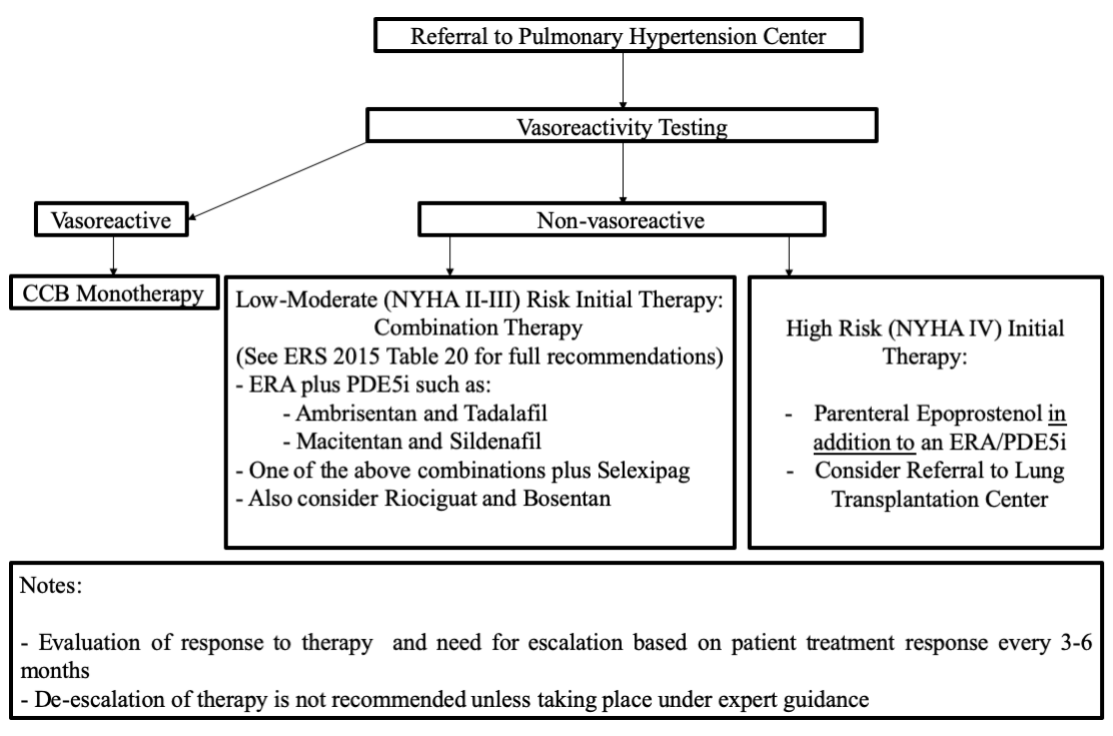
Proposed algorithm for treatment of pulmonary arterial hypertension based on 6th World Symposium on Pulmonary Hypertension recommendations. CCB, calcium channel blocker; ERA, endothelin receptor antagonist; NYHA, New York Heart Association; PDE5i, phosphodiesterase inhibitor.

Treatment of WHO group 1 PAH by targeting the nitric oxide, endothelin, and prostaglandin pathways has been standard since the 2003 Venice WSPH guidelines; no drugs targeting other pathways have been approved in the interim. The 6th WSPH task force has proposed a revised treatment algorithm ( Figure 1) that can be summarized as follows:

- All patients with a diagnosis of WHO group 1 PAH should be referred to a PH center to guide treatment and ongoing management.
- Monotherapy from any class based on suitability has been relegated a “residual role” in patients with the following:
  1) Vasoreactive PH patients who maintain reactivity, functional class I/II with sustained hemodynamic improvement after at least 1 year on calcium channel blockers only
  2) Patients with a low-risk profile who have historically been stable on monotherapy
  3) IPAH patients more than 75 years old with multiple risk factors for left heart disease
  4) PAH patients with suspicion or high probability of PVOD or PCH
  5) Patients with PAH associated with HIV, portopulmonary hypertension, or uncorrected congenital heart disease, as they were not included in RCTs of upfront combination therapy
  6) Patients with very mild disease defined on the basis of WHO functional class I, PVR of 3–4 WU, mPAP of less than 30 mm Hg, and normal right ventricle at echocardiography
  7) Combination therapy unavailable or contraindicated.
- Intravenous epoprostenol received the strongest recommendation for therapy in high-risk patients because of proven mortality benefit in patients with PAH even as monotherapy.
- The following dual-combination therapies are recommended on the basis of evidence:
  1) Macitentan and sildenafil
  2) Riociguat and bosentan
  3) Selexipag and endothelin receptor antagonist (ERA) or phosphodiesterase inhibitor (PDE5i) or both
- Transition from one therapy to another thought to be clinically as efficacious should be carried out under expert guidance.
- Given the lack of data and risk of decompensation, “stepping down” of therapy because of significant clinical improvement is generally advised against and should be carried out under expert guidance.
- If lack of clinical efficacy is noted with selexipag or non-parenteral prostacyclins, transition to parenteral prostanoids is recommended.

As data have accumulated from RCTs over the past decade, an increasing emphasis is being placed on upfront combination therapy. Longer-term data are needed to assess the cumulative impact of this strategy on long-term survival. With the drop in the mPAP criterion from 25 to 20 mm Hg, it is imperative that well-designed clinical trials be carried out to determine the impact on long-term outcomes with early intervention.

## Conclusions

Convincing epidemiological data have provided a rationale that justifies a revision in the hemodynamic definition of PH as mPAP of more than 20 mm Hg ^14^. Importantly, new clinical trials have led to an updated treatment algorithm for patients with group 1 PH, in which upfront combination therapy in treatment-naïve patients is now a recommended option ^32, 33^. Given the improved sophistication of our clinical data and genomic understanding, the care of patients with HPAH has entered an era of simultaneously discovering more about the complex genetic underpinnings that lead to mixed penetrance and optimizing treatment for those affected. In conclusion, the 6th WSPH has ushered in new clinical management paradigms while integrating ever-advancing scientific knowledge into the care of the patient with PH.

## Abbreviations

6MWD, six-minute walk distance; CpcPH, combined pre- and post-capillary pulmonary hypertension; HPAH, hereditary pulmonary arterial hypertension; IPAH, idiopathic pulmonary arterial hypertension; IpcPH, isolated post-capillary pulmonary hypertension; mPAP, mean pulmonary artery pressure; PAH, pulmonary arterial hypertension; PAWP, pulmonary arterial wedge pressure; PCH, pulmonary capillary hemangiomatosis; PH, pulmonary hypertension; PVOD, pulmonary veno-occlusive disease; PVR, pulmonary vascular resistance; RCT, randomized control trial; WES, whole exome sequencing; WGS, whole genome sequencing; WHO, World Health Organization; WSPH, World Symposium on Pulmonary Hypertension; WU, Wood units
